# Child assessments of vegetable preferences and cooking self-efficacy show predictive validity with targeted diet quality measures

**DOI:** 10.1186/s40795-019-0286-7

**Published:** 2019-03-02

**Authors:** Melissa Pflugh Prescott, Barbara Lohse, Diane C. Mitchell, Leslie Cunningham-Sabo

**Affiliations:** 1 Department of Food Science and Human Nutrition, University of Illinois at Urbana-Champaign, 905 S. Goodwin Ave, MC-182, Urbana, IL 61801-3896 USA; 2 Wegmans School of Health and Nutrition, Rochester Institute of Technology, Rochester, USA; 3 Department of Nutritional Sciences, Pennsylvania State University, Pennsylvania, USA; 4 Department of Food Science and Human Nutrition, Colorado State University, Colorado, USA

**Keywords:** Cooking, Youth, Diet assessment, Vegetable preference, Diet quality

## Abstract

**Background:**

Cooking interventions have the potential to improve child diet quality because cooking involvement is associated with positive changes in dietary behavior. Valid and reliable instruments that are low-cost and convenient to administer are needed to feasibly assess the impact of cooking interventions on dietary behavior. The purpose of the current research is to examine the validity of fruit and vegetable preferences, cooking attitudes and self-efficacy assessments to predict targeted Healthy Eating Index-2010 (HEI) scores among 4th-grade youth.

**Methods:**

Child fruit and vegetable preferences, cooking attitudes, self-efficacy, age, sex and race/ethnicity were collected with the *Fuel for Fun* survey in classroom settings using a standardized administration protocol. Child dietary assessment data consisted of three 24-h dietary recalls collected by telephone over a 2–4 week period by trained interviewers using a standard protocol. Bootstrapped linear regressions examined the predictive validity of fruit and vegetable preference, cooking attitudes and cooking self-efficacy for the Total and 4 targeted HEI components: whole fruit, total vegetables, green vegetables and beans, and empty calories. Logistic regressions were used to confirm the relationships between *Fuel for Fun* survey items and HEI components. Sex and a categorical variable for race/ethnicity were included as a priori controls in each regression model.

**Results:**

Vegetable preference predicted positive associations with HEI Total Score, Total Vegetables, and Green Vegetables and Beans (*p* < 0.05) Each additional 2 point increase in cooking self-efficacy was associated with a 1.33 point HEI Score increase, even after including BMI z-score as a control (b = 0.667, *p* = 0.003). Fruit preference and cooking attitudes did not significantly predict HEI total or component scores.

**Conclusions:**

This study provides evidence that low-cost, validated measures of vegetable preferences and cooking self-efficacy predict diet quality in 4th grade children. These results also reinforce the relationship between cooking and healthful dietary behavior.

## Background

The diet quality of U.S. children remains an important public health concern. Even though solid fat and added sugar intakes have significantly decreased from 2003 to 2014 among 6–11 year olds [1], the vast majority of American children exceed the recommended intake for empty calories and fail to meet the recommendations for total vegetables, total fruit and dark green vegetables [2]. The 2015 Dietary Guidelines for Americans encourages increasing vegetable and fruit intake and reducing the consumption of solid fats and added sugars. Furthermore, higher intakes of vegetables and fruit were the only diet characteristics that were consistently identified in the Advisory Committee’s investigation of the associations between dietary patterns and key beneficial health outcomes in the 2015 Dietary Guidelines for Americans Advisory Report [3].

Cooking interventions have the potential to improve child diet quality because cooking involvement is associated with positive changes in dietary behavior [4–8]. Canadian children who cook at home consume more fruits and vegetables, on average, compared to children who are not involved with cooking at home [5]. Children who participated in the LA Sprouts cooking and gardening intervention increased their fiber intake by 22%, compared to a 12% reduction among controls (*p* = 0.04) [9]. In the UK, 9 to 11 year old children had increased vegetable consumption and cooking confidence after participating in a culinary education intervention [4].

Valid and reliable instruments are required to accurately assess the impact of cooking interventions on dietary behavior and diet quality. A variety of dietary assessment tools can be used to comprehensively and quantitatively evaluate food and beverage intake but many of these measures are resource intensive [10] and often too expensive especially for larger samples [11]. The 24-h recall is considered the least biased of the self-reported methods [12] and is generally considered the best method for assessing children’s diets. Regardless of the method, the challenges and cost associated with detailed quantitative measures of child diet to assess diet quality suggest a need for more feasible proxy measures that could be administered in studies with large samples. Any proxy measure of child diet quality would need to validated relative to gold-standard reference methods such as multiple pass 24-h recalls [4]. Criterion-related validity assesses how closely scores from a new measure relate to the scores from the reference measure. Predictive validity, one form of criterion validity, is used when scores from the new measure are used to predict the scores on a criterion measure that is administered at a later point in time, rather than concurrently [13].

Fruit and vegetable preference [6, 8, 14, 15], cooking attitudes [14, 15], and cooking self-efficacy [4, 6, 14, 15] are common outcome measures for child cooking interventions that can be feasibly collected using validated and reliable tools and would be appropriate to test as potential proxies for diet quality. According to research completed in 1996 by Domel et al and 1997 by Resnicow et al, fruit and vegetable preferences significantly predict fruit and vegetable consumption, [16, 17]. No studies have examined the predictive validity of fruit and vegetable preference for diet quality relative to 24 h dietary recalls, and the relationships between cooking attitudes and cooking self-efficacies and diet quality are not well understood.

The purpose of this cross-sectional study was to examine the validity of fruit and vegetable preferences (FP, VP, FVP), cooking attitudes (AT) and self-efficacy (SE) assessments to predict diet quality among Colorado 4th-graders participating in *Fuel for Fun: Cooking with Kids Plus Parents and Play* (FFF).

## Methods

### Setting and participants

FFF is a cooking-focused, classroom-based intervention to improve 4th-grade student’s dietary attitudes and behaviors by increasing their interest and skills in preparing and tasting a wide variety of fruit and vegetable-rich dishes [18]. A total of 1409 students across 8 public elementary schools from 2 school districts in the same Northern Colorado county participated in FFF, and 996 of these students were offered the opportunity to participate in dietary assessment.

### Data collection and measures

All data were collected at study baseline when the child was in 4th grade. Child FVP, FP, VP, AT, SE, age, sex and race/ethnicity were collected with the *Fuel for Fun* survey in classroom settings using a standardized administration protocol [18]. FP and VP were assessed using 7 fruit and 11 vegetable preference questions, respectively. Each item had 5 response options, scored from 1 to 5. Scores of 7–35 were possible for FP and 11–55 for VP. FVP preference scores, determined by the sum of the fruit and vegetable preference scores, could range from 18 to 90. A 3 point increase in FVP is clinically significant [18]. Six items each with 5 response options, assessed cooking attitudes; possible scores ranged from 6 to 30. Eight items with 5 response options estimated cooking self-efficacy, with possible scores ranging from 8 to 40. A two point change in cooking attitudes or self-efficacy is considered clinically significant [18]. All items were previously confirmed for face and content validity and test-retest reliability [19].

After survey completion, *Fuel for Fun* survey participants were recruited to participate in dietary assessment. Child dietary assessment data consisted of three 24-h dietary recalls collected by telephone over a 2–4 week period by trained interviewers using a standard protocol [18]. Data were collected using Nutrition Data System for Research (NDSR) software version 2013 and 2014 (Nutrition Coordinating Center, University of Minnesota). Unannounced telephone interviews (approximately 20 min in duration) were conducted with both child and parent present on randomly selected days, with the goal of obtaining 2 weekdays and 1 weekend recalls. Only students with > 2 dietary recalls were included in this study to reduce random error associated with day-to-day dietary intake variability.

Diet quality is assessed using the Healthy Eating Index 2010 (HEI) [20]. The HEI is a valid and reliable diet quality measure that evaluates compliance with key recommendations from the Dietary Guidelines for Americans. The HEI is comprised of 12 component scores that are summed together to generate a total HEI-2010 score (possible range 0 to 100). This study will focus on the total HEI, as well as 4 of the 12 component scores: whole fruit (possible range 0 to 5), total vegetables (possible range 0 to 5), greens and beans (possible range 0 to 5), and empty calories (possible range 0 to 20). Higher HEI scores signal a diet that aligns better with dietary recommendations [20].

Height and weight were measured by trained personnel using a standard protocol on the same day as survey administration. Height was measured to the nearest tenth of a centimeter using a portable stadiometer (SECA, Model 213) and weight measured to the nearest tenth of a kilogram using a standard scale (Health o meter, Model 394KLX). As part of the protocol youth were instructed to remove their shoes and heavy clothing prior to measurement.

### Data analysis

Health Eating Index 2010 scores were calculated from Nutrition Data Systems for Research generated output files for nutrients and food groups. Food group data from output files were converted from servings to ounce and cup equivalents and then expressed on per 1000 kcal basis. Criteria for minimum and maximum scores were used to create formulas to calculate 12 individual components scores and summed to calculate total HEI scores [21].

Predictive validity of FV preference, FP, VP, cooking AT and cooking SE for the Total and these 4 targeted HEI components were examined using linear regression analyses. The large number of HEI component scores at the upper and/or lower bounds required bootstrapping of linear regressions and logistic regressions. Logistic regressions were used to confirm the relationships between FFF survey items and meeting the maximum score for HEI components. Sex and a categorical variable for race/ethnicity were included as a priori controls in each regression model. Each model was run a second time (Model 2) with the inclusion of BMI z-score as a covariate because it was a confounder. A *p*-value of less than 0.05 was considered statistically significant for linear regressions, and odds ratio (OR) 95% confidence intervals that did not cross 1.0 were considered statistically significant for logistic regressions.

## Results

A total of 108 students completed at least one dietary recall, and 101 completed at least 2 dietary recalls. Of the 101 children who completed > 2 dietary recalls, 73% had one weekend day included in their dietary assessment. Differences in race/ethnicity, sex, or age between participants who completed only 1 dietary recall (*n* = 7) and those who completed 2 or more recalls (*n* = 101) were not significant, but participants completing only 1 recall had significantly higher SE (*p* = 0.050), AT (*p* = 0.026) and FP scores (*p* = 0.052). Children who had recall heterogeneity in day type (i.e. included both weekend and weekdays) did not differ significantly in any of the targeted HEI outcomes nor the FFF survey measure predictor variables from those children with only weekday or weekend day recalls. Participants (*n* = 101) were predominately white, non-Hispanic and at a healthy weight (mean BMI percentile 49.4 + 30.84, range 1.0 to 99.0). Demographic results, as well as student VP, FP, cooking AT and cooking SE are similar to youth in previous samples [15, 22]. Total HEI scores (possible range 0–100) ranged from 33.8–91.1 (Table 1). HEI or survey scores did not differ by sex, with the exception of whole fruit score, which was greater in boys (*p* = 0.04) (Table 1).

**Table 1:** Characteristics of Fourth-Grade Students (*n* = 101)

	*n*	(%)		*n*	*Mean*	±	*SD*
Female	47	(46.5)	Age (years)	101	9.1	±	0.4
Ethnicity	101		Fruit and Vegetable Preference	98	65.6	±	11.3
White	79	(78.2)	Fruit Preference	101	29.1	±	4.5
Hispanic	13	(12.8)	Vegetable Preference	98	36.4	±	8.0
Black	1	(1.0)	Cooking Attitude	101	26	±	3.4
Asian	3	(3.0)	Cooking Self-Efficacy	100	33.7	±	5.9
American Indian	2	(2.0)	Healthy Eating Index – 2010 (HEI)	101	62.5	±	10.7
Two or more races	3	(3.0)	Whole Fruit Component	101	3.6	±	1.6
BMI Category	93		Total Vegetable Component	101	2.3	±	1.5
Underweight	5	(5.4)	Greens and Beans Component	101	1.3	±	1.7
Healthy Weight	72	(77.4)	Empty Calories Component	101	18.8	±	2.0
Overweight	5	(5.4)					
Obese	11	(11.8)					

*SD = Standard Deviation, BMI = Body Mass Index*

Possible ranges of survey items: fruit and vegetable preference (18–90), fruit preference (7–35), vegetable preference (11–55), cooking attitude (6 to 30), and cooking self-efficacy (8 to 40)

Possible HEI score range is 0–100, Whole Fruit Component ranges from 0 to 5, Total Vegetable Component ranges from 0 to 5, Green Vegetables and Beans Component ranges from 0 to 5, and Empty Calories Component ranges from 0 to 20

VP positively predicted HEI total, total vegetables, and greens and beans scores (*p* < 0.05). The relationship between VP and total vegetable score tended toward significance once BMI z-score was added to the model (*p* = .09; Table 2). Each additional 2 point increase in cooking SE was associated with a 1.33 point HEI Score increase, even after including BMI z-score as a control (b = 0.667, *p* = 0.003). Cooking SE also positively predicted total vegetable score (*p* = 0.008) and Whole Fruit (*p* = 0.031). The positive associations between cooking SE and whole fruit score persisted when BMI z-score was controlled. Cooking SE also predicted total vegetable score (p = 0.008) and tended toward significance in Model 2 (*p* = 0.059). FP and AT did not significantly predict HEI total or component scores.

**Table 2:** Predicted change in Healthy Eating Index Score and Component Scores associated with changes in fruit and vegetable preference, cooking attitude, and cooking self-efficacy scores among 4th grade children

Predictor Variable	Model 1^a^		Model 2^b^	
	*b*	*SE(b)*	*b*	*SE(b)*
	*Outcome Variable* ^c^			
	*Healthy Eating Index – 2010 (HEI)*			
Fruit and Vegetable Preference^d^	0.172	0.092	0.178	0.109
Fruit Preference^e^	− 0.107	0.232	−0.179	0.283
Vegetable Preference^f^	**0.361**	**0.129**	**0.392**	**0.153**
Cooking Attitude^g^	0.648	0.353	0.609	0.398
Cooking Self-Efficacy^h^	**0.595**	**0.172**	**0.667**	**0.209**
	*HEI Whole Fruit Component*			
Fruit and Vegetable Preference	0.023	0.014	0.021	0.016
Fruit Preference	0.036	0.036	0.019	0.039
Vegetable Preference	0.033	0.02	0.034	0.022
Cooking Attitude	0.099	0.052	0.091	0.057
Cooking Self-Efficacy	**0.061**	**0.029**	**0.07**	**0.035**
	*HEI Total Vegetable Component*			
Fruit and Vegetable Preference	**0.032**	**0.012**	0.024	0.014
Fruit Preference	0.055	0.029	0.039	0.034
Vegetable Preference	**0.044**	**0.017**	0.034	0.020
Cooking Attitude	0.034	0.045	0.015	0.046
Cooking Self-Efficacy	**0.059**	**0.022**	0.052	0.027
	*HEI Greens and Beans Component*			
Fruit and Vegetable Preference	**0.032**	**0.014**	0.03	0.017
Fruit Preference	0.008	0.037	0.009	0.042
Vegetable Preference	**0.060**	**0.019**	**0.054**	**0.023**
Cooking Attitude	−0.014	0.056	−0.022	0.062
Cooking Self-Efficacy	0.028	0.025	0.014	0.032
	*HEI Empty Calories Component*			
Fruit and Vegetable Preference	0.028	0.024	0.018	0.026
Fruit Preference	0.019	0.05	−0.028	0.047
Vegetable Preference	0.049	0.036	0.043	0.041
Cooking Attitude	0.034	0.068	0.024	0.066
Cooking Self-Efficacy	0.133	0.058	0.120	0.070

^a^ Linear regression adjusted for race/ethnicity and gender

^b^ Linear regression adjusted for race/ethnicity, gender, and BMI z-score

^c^ Total HEI score ranges from 0 to 100 points. HEI Whole Fruit, Total Vegetable, and Greens and Beans ranges from 0 to 5 points. HEI Empty Calories ranges from 0 to 20 points

^d^ For all outcomes: Model 1 n = 98, Model 2 n = 90; possible scores ranged from 18 to 90. Higher scores indicated greater preference

^e^ For all outcomes: Model 1 n = 101, Model 2 n = 93; possible scores ranged from 7 to 35. Higher scores indicated greater preference

^f^ For all outcomes: Model 1 *n* = 98, Model 2 *n* = 90; possible scores ranged from 11 to 55. Higher scores indicated greater preference

^g^ For all outcomes: Model 1 *n* = 101, Model 2 *n* = 93; possible scores ranged from 6 to 30. Higher scores indicated more positive cooking attitudes

^h^ For all outcomes: Model 1 n = 100, Model 2 n = 92; possible scores ranged from 8 to 40. Higher scores indicated greater cooking self-efficacy

**Bold** indicates statistical significance with p < 0.05

*b* = Regression Coefficient, SE = Standard Error, BMI = Body Mass Index

The relationships between Fuel for Fun survey items and targeted HEI components derived from linear regressions were largely consistent with the results from the logistic regression analysis (Table 3). VP and cooking SE positively predicted total vegetable score. VP also positively predicted greens and beans score. The relationship between cooking SE and Green Vegetables and Beans was also positive, but not significant once BMI z-score was added to the model. In addition to linear regression results confirmation, the relationship between VP and whole fruit score was also significant, which can be extrapolated to a 3 point increase in VP. A 3 point increase in VP increased the odds of meeting whole fruit recommendations (i.e. consuming > 0.8 cup equivalents of whole fruit per 1000 kcal) by 30.9% (OR = 1.094, 95% CI: 1.024, 1.169).

**Table 3:** Adjusted logistic regression results for prediction of adherence to dietary guidelines recommendations by vegetable preference and cooking self-efficacy scores among 4th grade children

Predictor Variable	Model 1^a^			Model 2^b^		
		*95% CI*			*95% CI*	
	*OR*	*Lower*	*Upper*	*OR*	*Lower*	*Upper*
	*Outcome Variable* ^*c*^					
	*HEI Whole Fruit Component*					
Vegetable Preference^d^	**1.075**	**1.014**	**1.140**	**1.094**	**1.024**	**1.169**
Cooking Self-Efficacy^e^	1.067	0.985	1.156	1.096	0.999	1.202
	*HEI Total Vegetable Component*					
Vegetable Preference	**1.108**	**1.013**	**1.212**	**1.114**	**1.016**	**1.222**
Cooking Self-Efficacy	**1.191**	**1.007**	**1.408**	**1.222**	**1.021**	**1.464**
	*HEI Greens and Beans Component*					
Vegetable Preference	**1.084**	**1.020**	**1.152**	**1.070**	**1.002**	**1.142**
Cooking Self-Efficacy	**1.094**	**1.003**	**1.193**	1.062	0.967	1.166
	*HEI Empty Calories Component*					
Vegetable Preference	1.022	0.971	1.075	1.024	0.968	1.083
Cooking Self-Efficacy	1.028	0.956	1.106	1.035	0.951	1.127

^a^ Logistic regression adjusted for race/ethnicity and gender

^b^ Logistic regression adjusted for race/ethnicity, gender, and Body Mass Index z-score

^c^ Binary variable (yes/no) indicating status of scoring the maximum score for each HEI component: HEI Whole Fruit (5 point max), Total Vegetable (5 point max), Greens and Beans (5 points), and Empty Calories (20 points). Scoring the maximum score indicates conformance to the Dietary Guidelines for Americans

^d^ For all outcomes: Model 1 *n* = 98, Model 2 *n* = 90; possible scores ranged from 11 to 55. Higher scores indicated a greater cooking self-efficacy

^e^ For all outcomes: Model 1 *n* = 100, Model 2 *n* = 92; possible scores ranged from 8 to 40. Higher scores indicated a greater vegetable preference

**Bold** indicates statistical significance (i.e. 95% confidence intervals that do not cross 1.00)

OR = Odds Ratio, CI = Confidence Interval, HEI = Healthy Eating Index 2010

## Discussion

The VP and SE assessments utilized in *FFF* are consistent with dietary quality as assessed by HEI indicators generated via multiple pass 24-h recalls, demonstrating predictive validity of these *Fuel for Fun* outcome measures. These results also contribute to the evidence of the positive relationship between child dietary preferences and dietary behaviors. This link was previously demonstrated based upon estimates of intakes derived from 7-day food records [16, 17] and food frequency questionnaires [23]; the current study arrived at similar conclusions using a more robust diet assessment technique. However, previous studies established relationships between both fruit and vegetable preferences and consumption behaviors [16, 17, 23]. Yet, the current study found a significant relationship between vegetable preference and overall HEI score, but fruit preference did not significantly predict any of the targeted HEI outcomes. This may be because of children’s inherent tendency for high fruit preferences, [24–26] which may limit the ability of fruit preference to be a proxy for overall diet quality.

Our findings that cooking SE predicts Total HEI, Total Vegetable and Whole Fruit scores extends the authors’ conclusions from a previous study, that reported prior cooking involvement predicts changes in fruit and vegetable preferences after a cooking intervention [15]. Additionally, the relationship between cooking SE and diet quality is consistent with the results of Caraher and colleagues and Jarpe-Ratner and colleagues. Both studies concluded that cooking SE and vegetable consumption significantly increased after school-based cooking interventions, but both of these studies used a screener rather than dietary recalls to measure vegetable intake [4, 6]. In the current study, we estimate that for every 3 point increase in cooking self-efficacy we expect a 82.5% increase in total vegetable score (OR = 1.222, 95% CI: 1.021–1.464), suggesting a positive relationship between child cooking self-efficacy and meeting the dietary guidelines for total vegetable consumption. However, the study design does not establish the temporality of this relationship.

Additional limitations include a relatively small sample size and challenges with dietary assessments. Domel et al’s 1996 assessment and Resnicow et al’s 1997 assessment of the relationship between FVP and fruit and vegetable consumption featured 392 and 1398 participants, respectively. However, the dietary intake of the 108 students included in our study was similar to national averages [21], suggesting generalizability of our results. The challenges of child dietary assessment have been previously discussed [10, 27, 28]. In addition, child diet assessment methods, including 24-h recalls, have been infrequently validated [27]. Having a parent present during the 24-h recall telephone interviews may have imparted some social desirability bias, but parent influence was helpful in reminding their children of forgotten foods and portion sizes. The 24-h, multiple pass recall method is the best diet assessment available, but the method has inherent limitations, such as systematic and random error [12]. However, all students in the sample had two or more dietary recalls, reducing the influence of the random error associated with day-to-day variation in dietary intake [12]. Dietary recall invitations were planned so that diet assessments included weekday as well as weekend day intake. Both weekday and weekend day recalls occurred for 73% of participants. However, for any of the targeted HEI outcomes or FFF survey measure predictor variables the differences were not significant between children who had heterogeneity in day type (i.e. recalls included both weekend and weekdays) vs. those with only weekday or weekend day recalls. Lastly, the study sample had limited racial or ethnic diversity. Future studies are needed to assess the predictive validity of these measures in more diverse populations.

This study has several strengths. The majority of results were robust across two different analyses methods. Our sample had a slightly higher mean HEI (62.5 + 10.7) and Green Vegetables and Beans HEI component score (1.3 + 1.7) compared to the national average (55.1 + 0.72 and 07.0 + 0.09, respectively), but total vegetable and whole fruit scores were very similar to the national average [21]. In addition, we used the multiple pass interviewer-administered 24-h dietary recall method conducted by a skilled dietary assessment center, and the parents were present for most of the dietary assessments. The FVP, AT, and SE assessment tools are reliable and have been previously validated.

The study findings have important implications. The predictive validity of the FFF outcome measurements suggests that using these VP and SE assessments may be particularly useful to researchers and practitioners when indicators of diet quality are needed but constraints of time or cost abrogate the use of dietary assessment methods. The positive, predictive relationship between cooking self-efficacy and diet quality also supports the use of cooking interventions to improve overall diet quality, vegetable consumption, and whole fruit consumption. Similarly, the significant relationship between vegetable preference and overall diet quality suggests that interventions to improve child vegetable preferences are warranted.

## Conclusion

This study provides evidence that low-cost measures of vegetable preferences and cooking self-efficacy predict diet quality in 4th grade children. These easy to administer, valid and reliable instruments encompass constructs common to many cooking interventions, increasing their value as evaluation tools. These results also reinforce the relationship between cooking and healthful dietary behavior.

## Abbreviations

AT: cooking attitude
BMI: Body Mass Index
FFF: *Fuel for Fun*
FP: fruit preference
FVP: fruit and vegetable preference
HEI: Healthy Eating Index- 2010
SE: cooking self-efficacy
VP: vegetable preference
